# The Role of Acyl-CoA Synthetase 1 in Bioactive Lipid Accumulation and the Development of Hepatic Insulin Resistance

**DOI:** 10.3390/nu16071003

**Published:** 2024-03-29

**Authors:** Piotr Zabielski, Monika Imierska, Kamila Roszczyc-Owsiejczuk, Mariusz Kuźmicki, Paweł Rogalski, Jarosław Daniluk, Agnieszka U. Błachnio-Zabielska

**Affiliations:** 1 Medical Biology Department, Medical University of Bialystok, 15-222 Bialystok, Poland; piotr.zabielski@umb.edu.pl; 2 Hygiene, Epidemiology and Metabolic Disorders Department, Medical University of Bialystok, Mickiewicza 2c, 15-222 Bialystok, Poland; monika.imierska@umb.edu.pl (M.I.); kamila.roszczyc-owsiejczuk@umb.edu.pl (K.R.-O.); 3 Gynecology and Gynecological Oncology Department, Medical University of Bialystok, 15-276 Bialystok, Poland; mariusz.kuzmicki@umb.edu.pl; 4 Gastroenterology and Internal Medicine Department, Medical University of Bialystok, 15-276 Bialystok, Poland; pawel.rogalski@umb.edu.pl (P.R.); jaroslaw.daniluk@umb.edu.pl (J.D.)

**Keywords:** liver, insulin resistance, obesity, high-fat diet, lipids, gene silencing, mass spectrometry

## Abstract

The liver plays a crucial role in glucose metabolism. Obesity and a diet rich in fats (HFD) contribute to the accumulation of intracellular lipids. The aim of the study was to explore the involvement of acyl-CoA synthetase 1 (ACSL1) in bioactive lipid accumulation and the induction of liver insulin resistance (InsR) in animals fed an HFD. The experiments were performed on male C57BL/6 mice divided into the following experimental groups: 1. Animals fed a control diet; 2. animals fed HFD; and 3. HFD-fed animals with the hepatic ACSL1 gene silenced through a hydrodynamic gene delivery technique. Long-chain acyl-CoAs, sphingolipids, and diacylglycerols were measured by LC/MS/MS. Glycogen was measured by means of a commercially available kit. The protein expression and phosphorylation state of the insulin pathway was estimated by Western blot. HFD-fed mice developed InsR, manifested as an increase in fasting blood glucose levels (202.5 mg/dL vs. 130.5 mg/dL in the control group) and inhibition of the insulin pathway, which resulted in an increase in the rate of gluconeogenesis (0.420 vs. 0.208 in the control group) and a decrease in the hepatic glycogen content (1.17 μg/mg vs. 2.32 μg/mg in the control group). Hepatic ACSL1 silencing resulted in decreased lipid content and improved insulin sensitivity, accounting for the decreased rate of gluconeogenesis (0.348 vs. 0.420 in HFD_(+/+)_) and the increased glycogen content (4.3 μg/mg vs. 1.17 μg/mg in HFD_(+/+)_). The elevation of gluconeogenesis and the decrease in glycogenesis in the hepatic tissue of HFD-fed mice resulted from cellular lipid accumulation. Inhibition of lipid synthesis through silencing ACSL1 alleviated HFD-induced hepatic InsR.

## 1. Introduction

The liver, besides skeletal muscle and adipose tissue, plays a crucial role in glucose and lipid metabolism. Obesity and a diet rich in fats (HFD) lead to an excessive elevation of plasma free fatty acid (FFA) and the ectopic accumulation of intracellular lipids, which is associated with occurrence of cardiovascular diseases, insulin resistance (InsR), and type 2 diabetes (T2D) [1,2]. In the physiological state, insulin stimulates hepatic lipogenesis and glycogen synthesis and decreases the plasma blood glucose by inhibiting hepatic gluconeogenesis and glycogenolysis [3]. Upon intracellular translocation, fatty acids (FA) are activated to a long-chain acyl-CoAs (LCACoAs) by a long-chain acyl-CoA synthetase (ACSL). Subsequently, LCACoAs can be directed toward mitochondrial oxidation or be used as a main substrate for the synthesis of other intracellular lipids [4]. So far, five isoforms of ACSL have been identified. Members of the ACSL family play distinct roles in FA metabolism, accounting for unique substrate preferences and tissue distribution. ACSL1 is a predominant isoenzyme of ACSLs expressed in the hepatic tissue [5]. The content of LCACoAs has been demonstrated to increase in the livers of obese or HFD-fed rats [6,7,8]. Since LCACoAs are used for the de novo lipid synthesis, those compounds may indirectly inhibit insulin signaling cascade by participating in the synthesis of ceramides (Cer) and diacylglycerols (DAG). The intracellular accumulation of those biologically active lipids may lead to the induction of insulin resistance [9,10,11].

In the liver, insulin suppresses gluconeogenesis through the phosphorylation-dependent signaling cascade. Ligation of the insulin receptor (IR) results in activation of phosphorylation cascade, namely, IR receptor autophosphorylation; tyrosine phosphorylation of the IRS; and sequential activation of phosphatidylinositol 3-kinase (PI3-K) [12], atypical protein kinase C (aPKC), and protein kinase B (Akt/PKB) [13,14]. Akt/PKB is responsible for the inhibitory phosphorylation of the forkhead box O1 (FOXO1) transcription factor that regulates the expression of glucose-6-phosphatase (G6Pase) and phosphoenolpyruvate carboxykinase (PEPCK). Decreases in the expression of those key gluconeogenic enzymes leads to the suppression of gluconeogenesis [15,16]. Any decrease in Akt/PKB phosphorylation (activity) is often associated with an increase in the ceramide level, which activates phosphatase 2A (PPA2) responsible for the Akt/PKB dephosphorylation [17]. In an insulin-resistant state, insulin is unable to suppress the expression/activity of the aforementioned enzymes. In addition, insulin controls glucose metabolism by promoting glycogen synthesis. This regulation is mediated by Akt/PKB kinase, which phosphorylates and inactivates glycogen synthase kinase 3 (GSK3) [18], the enzyme that controls the activity of glycogen synthase 2 (GYS2). GYS2 is inhibited by phosphorylation and activated by glucose-6-phosphate (G6P) [19,20]. GYS2 and glycogen phosphorylase are, respectively, responsible for the synthesis and breakdown of hepatic glycogen. Insulin-mediated dephosphorylation regulates the activities of both enzymes [21]. Hepatic lipid accumulation is commonly accepted as a key constituent of hepatic InsR, but despite extensive research, the molecular mechanisms of hepatic InsR still remain incompletely understood. The aim of this study was to explain the role of ACSL1, particularly in terms of bioactive lipid accumulation and the development of hepatic InsR in animals fed an HFD. Since ACSL1 is the predominant hepatic isoform responsible for the synthesis of LCACoAs, and indirectly for the synthesis of signaling lipids, we silenced hepatic ACSL1 to determine its role in the accumulation of the respective bioactive lipids and the induction of hepatic InsR in HFD-fed mice.

## 2. Materials and Methods

### 2.1. Animals and Study Design

Consent to conduct the experiment was granted by the appropriate governing body (IACUC equivalent, Lokalna Komisja Etyczna ds. Doświadczeń na Zwierzętach, Oczapowskiego 13/4 Street, 10-718, Olsztyn, Poland, Approval date 23 September 2020, approval number 48/2020). Experiments were performed on male C57BL/6 mice (approx. 20 g of body weight, Jackson Laboratory, Bar Harbor, ME, USA). Upon arrival from Jackson Laboratory (Bar Harbor, ME, USA), mice were weighed and assigned randomly to cages. The sample size and power analysis, using our previous data regarding HOMA-IR parameters and the hepatic content of TAG and DAG, revealed the optimum group size of 8 (*p* < 0.05 and 90%) whereas the literature data showed *n*-numbers between 6 and 20 subjects per group. Animals were housed in typical conditions (21 °C ± 2, 12 h light/12 h dark cycle) with ad libitum access to water and an appropriate diet. After one week of acclimatization, the animals were randomly divided into following groups: the non-silenced control group (control, *n* = 10), fed a control low-fat diet ad libitum (Research Diets INC D12450J; 10% of fat calories, Research Diets, New Brunswick, NJ, USA) which was transfected with scrambled shRNA plasmid; the non-silenced high-fat diet group (HFD_(+/+)_, *n* = 10), housed on a diet rich in fats (Research Diets INC D12492; 60% of fat calories, Research Diets, New Brunswick, NJ, USA) and also transfected with non-coding shRNA plasmid; and the silenced high-fat diet group (HFD_(−ACSL1)_, *n* = 13), transfected with coding, functional shRNA plasmid. In all animal groups, shRNA-mediated plasmid transfection was performed using a hydrodynamic gene delivery (HGD) technique at the 6th week of feeding. Both the non-silenced LFD-fed control and HFD-fed, non-silenced HFD_(+/+)_ animals underwent the HGD procedure, but with non-coding, scrambled shRNA plasmid. All groups were subsequently housed on the appropriate diet for additional 2 weeks, for a total of 8 weeks of feeding at the end of experiment. Animals underwent glucose tolerance tests (OGTTs) one week before sacrifice. Exclusion criteria at the time of euthanasia included visible ataxia, decreased body weight, any visible wounds or sores, and signs of general distress. As the HGD and other procedures were well tolerated, there were no drop-outs in the study groups. On the day of euthanasia, animals received 2 h of infusion of D-[UL-^13^C_6_] glucose (Merck, Lebanon, NJ, USA) through the lateral tail vein at a rate of 5 nmol/min/g of body weight. Fifteen minutes prior to the infusion completion, 0.5 U/kg of insulin was administered through the infusion line. At the end of the experiment, liver samples were harvested, frozen in LN_2_, and stored at −80 °C for further analysis.

### 2.2. Plasmids and In Vivo shRNA Plasmids Injection

**Figure 1:**
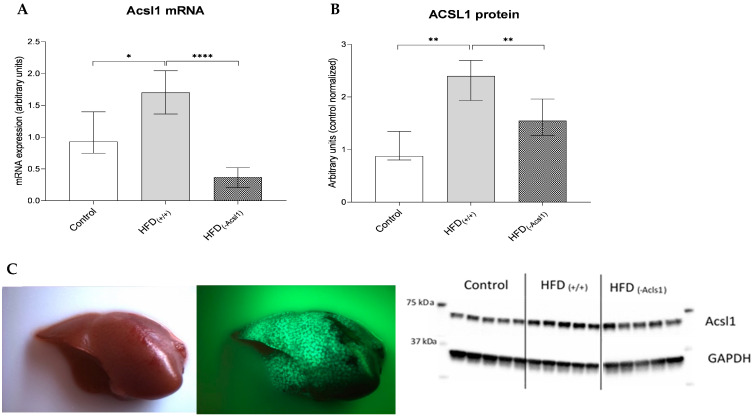
mRNA of Acsl1 in liver (**A**). Protein level of Acsl1 (**B**). Bars represent medians ± interquartile range; *—*p* < 0.05; **—*p* < 0.01; ****—*p* < 0.0001. Green fluorescent protein in liver after plasmid transfection: visible light of a mouse liver (photo on the left); TurboGFP fluorescence of isolated liver (photo on the right) (**C**). Fluorescence stereomicroscopy performed with the use of the Nightsea SFA-RB-GO fluorescence adapter (Nightsea, Hatfield, PA, USA)/DeltaPix Invenio 5SIII CMOS camera (DeltaPix, Smorum, Denmark).

Bacterial cultures expressing appropriate coding and scrambled shRNA plasmids were obtained from Horizon Discovery (previously Dharmacon, Cambridge, UK). Amplification and isolation of the plasmids was performed according to the manufacturer’s guidelines, with the use of GeneJET Plasmid Maxiprep Kit (Thermo Fisher Scientific, Waltham, MA, USA). A silencing mix was prepared using 3 different ACSL1 shRNA sequences (Set Cat. No.: V3SM11244-01EG14081), whereas scrambled shRNA (Cat. No.: VSC11708) was used for the non-silenced group. Gene silencing was performed after 6 weeks of feeding with the appropriate diet. Animals were anesthetized using ~2% isoflurane in oxygen (UNO BV, UNO, Zevenaar, The Netherlands). Hydrodynamic-based transfection was performed by injecting 100 μg of plasmids into Altogen’s liver in vivo transfection reagent (Altogen Biosystems, Las Vegas, NV, USA) via the lateral tail vein [22,23]. The volume of the transfectant mixture was adjusted with 150 mM PBS, pH = 7.2 to 10% of equivalent body weight (1 mL/10 g, 2.5 mL for a 25 g mouse, injected within 7 s). All plasmids encoded green fluorescent protein (GFP) as a marker of transfection efficiency (Figure 1C). The feeding with the appropriate diet continued for an additional 2 weeks, until the end of the experiment. The effect of silencing was noticeable just 24 h after the HGD procedure and lasted up to two weeks.

### 2.3. Lipid Measurements

#### 2.3.1. Sphingolipids

Hepatic sphingolipids levels were measured by UHPLC/MS/MS according to Blachnio-Zabielska et al. [24]. Liver tissue (approx. 15–20 mg) was homogenized in 250 mM sucrose, 25 mM KCl, 50 mM Tris, and 0.5 mM EDTA (pH = 7.4). Afterwards, appropriate ceramide internal standards were added (d18:1/C15:0-d7, d18:1/C16:0-d7, d18:1/C18:1-d7, d18:1/C18:0-d7, d17:1/20:0, d18:1/C24:1-d7, d17:1/20:0C24-d7), (Avanti Polar Lipids, Alabaster, AL, USA) and sphingolipids were extracted through sonication in isopropanol:water:ethyl acetate (30:10:60; *v*:*v*:*v*). After vortexing and centrifugation, supernatants were evaporated under N_2_ and dissolved in 2 mM ammonium formate/0.1% formic acid in methanol. Samples were resolved on Zorbax SB-C8 reversed-phase column 2.1 × 150 mm, 1.8 μm (Agilent Technologies, Santa Clara, CA, USA) using a binary gradient of 1 mM ammonium formate/0.1% formic acid in water (HPLC solvent A) and 2 mM ammonium formate/0.1% formic acid in methanol (HPLC solvent B). The sphingolipid analysis was performed in MRM mode (with multiple reaction monitoring) on a Sciex QTRAP 6500 + LC/MS/MS system (AB Sciex, Darmstadt, Germany).

#### 2.3.2. Diacylglycerols

Hepatic DAGs were quantified by UHPLC/MS/MS as described in Blachnio-Zabielska et al. [25]. DAG extraction was performed concurrently with sphingolipid extraction, using the same solvent system. Before extraction, a mixture of isotopically labeled internal standards (mixture I and II of deuterated DAG standards—Avanti Polar Lipids) was spiked into each homogenate. Chromatographic separation of DAG was performed on a 2.1 × 150 mm Zorbax SB-C8 column using the same solvent system as for sphingolipids. Individual DAG species were quantified in MRM mode on a Sciex QTRAP 6500 + LC/MS/MS system.

#### 2.3.3. Long-Chain Acyl-CoA

The level of hepatic LCACoAs was analyzed using UHPLC/MS/MS as described by Blachnio-Zabielska et al. [26]. LCACoA extraction was performed according to Minkler et al. [27] with the use of internal standards (C15:0-, 16:0(d4)-, C17-, C19:0-, C21:0-, C23:0- and 24:0(d4)-CoA) spiked into each sample. Samples were resolved on 2.1 mm × 150 mm Agilent ZORBAX Extend-C18, using a reverse-phase gradient of ammonium hydroxide (NH_4_OH) in water and NH_4_OH in ACN. The quantitative analysis of LCACOAs was performed in positive electrospray ionization mode (ESI+) on Sciex QTRAP 6500 + LC/MS/MS system (AB Sciex, Darmstadt, Germany).

#### 2.3.4. Plasma FFA, Hepatic Triacylglycerols and Liver Glycogen Content

The total plasma FFA concentration was estimated with the MAK044 colorimetric assay (Sigma-Aldrich, St. Louis, MO, USA). The total content of hepatic triacylglycerols was measured with the MAK264-1KT fluorometric kit (Sigma Aldrich, St. Louis, MO, USA). Hepatic glycogen was measured with the MAK016 Glycogen Assay Kit (Merck, Lebanon, NJ, USA).

### 2.4. Liver Glucose-6-Phosphate (G6P) Content and Estimation of Hepatic Gluconeogenesis

The tissue content of G6P was quantified by GC/MS according to Young et al. [28], with modifications. Hepatic gluconeogenesis was measured through plasma glucose isotopomer distribution analysis (MIDA) after infusion of stable-isotope labeled D-[UL-^13^C_6_]glucose [29,30]. Glucose isotopomer distribution was measured by means of GC/MS after conversion to di-O-isopropylidene propionate derivatives [31,32]. Isotopomer enrichment was calculated with the use of Python IsoCor v2 software, after correction for tracer purity (99% APE) [33]. The reciprocal pool model of the gluconeogenesis (GNG) by Haymond and Sunehag [34] and Chung et al. [35] was used for estimating the fractional gluconeogenesis (F_GNG_) and gluconeogenic rate (GNG).

### 2.5. Protein Expression

Liver proteins prepared in Laemmli buffer were resolved using SDS-PAGE on TGX pre-cast gels (Bio-Rad, Hercules, CA, USA) and transferred to a PVDF membrane via semi-dry transfer (Trans Blot Turbo, Bio-Rad, Hercules, CA, USA). Membranes were blocked with EveryBlot buffer (Bio-Rad Hercules, CA, USA) and incubated with the appropriate primary antibodies: ACSL1, Akt, Phospho-Akt (Ser473), GYS2, PEPCK, pFOXO1(Ser256), FOXO1, and GAPDH (Thermo Fisher Scientific, Waltham, MA, USA). After washing and incubation with an HRP-conjugated secondary antibody, protein bands were detected with an ECL chemiluminescent assay on a Bio-Rad ChemiDoc XRS+ imaging system (Bio-Rad, Hercules, CA, USA). Expression of the target protein was normalized to the GAPDH and expressed as fold changes over the control group value. Original blot pictures and their description are presented in Supplement Figures S1–S8.

### 2.6. Real-Time PCR

Liver RNA was isolated using a Total RNA Mini kit (A&A Biotechnology, Gdańsk, Poland). First-strand cDNA was synthesized with the TranScriba Kit (A&A Biotechnology, Gdańsk, Poland). Real-time PCR was performed by means of the LightCycler 480 SYBR Green I Master (Roche, Mannheim, Germany) using a LightCycler480 system (Roche, Mannheim, Germany). The nucleotide sequences of the forward and reverse primers for the target genes encoding ACSL1 and GAPDH (which was used as a housekeeping gene) were as follows (5′ to 3′): ACSL1 (forward): ACTGTGCAGGAACAAGGATAT, ACSL1 (reverse): TAAGTAAGGCAGTGTTCCGT; Glyceraldehyde-3-phosphate Dehydrogenase (GAPDH) (forward): AGGAGAGTGTTTCCTCGTCC, GAPDH (reverse): AGGAGACAACCTGGTCCTCA. GAPDH was used as reference gene and loading control.

### 2.7. Plasma Insulin and Glucose Concentration

Insulin concentration was measured using an ELISA insulin assay (Crystalchem, Elk Grove Village, IL, USA). Plasma glucose concentration was determined with an AccuChek Aviva glucometer (Roche, Mannheim, Germany).

### 2.8. OGTT

OGTT was performed on fasting animals. Blood samples were taken at 15, 30, 60, and 120 min after oral gavage of glucose at a dose of 2 g/kg of lean mass. Additionally, at 15 and 60 min, blood was collected for the estimation of insulin concentration.

### 2.9. Homeostatic Model Assessment for Insulin Resistance (HOMA-IR)

HOMA-IR was calculated with an equation modified for use in rodent-based studies [36]: HOMA-IR = [fasting glucose (mg/dL) × fasting insulin (μIU/mL)]/2430.

### 2.10. Protein Concentration

Protein content in homogenates was measured colorimetrically with a Pierce 660 nm protein assay kit (Thermo Fisher Scientific, Waltham, MA, USA) against a standard curve prepared on serum albumin in PBS.

### 2.11. Statistical Analysis

All values were expressed as medians and interquartile ranges (IQRs). The significance of the observed changes was determined using the non-parametric Mann–Whitney U test. A significance level of *p* < 0.05 was established. The statistical analysis was performed using GraphPad Software’s Prism 9.3.1.

## 3. Results

### 3.1. Effect of HFD Diet and Silencing on Hepatic Expression of ACSL1 Gene

The high-fat diet feeding significantly increased both the ACSL1 mRNA (by 49%, *p* < 0.05) and protein (over 2.5 times, *p* < 0.01) in the livers of the HFD_(+/+)_ animals as compared to the controls. Targeted silencing in HFD_(−ACSL1)_ animals contributed to the reduction in ACSL1 at the mRNA and protein levels by 72% (*p* < 0.0001) and 35% (*p* < 0.05), respectively, vs. HFD_(+/+)_ values (Figure 1).

### 3.2. Plasma FFA Concentration and Hepatic Lipid Content

**Figure 2:**
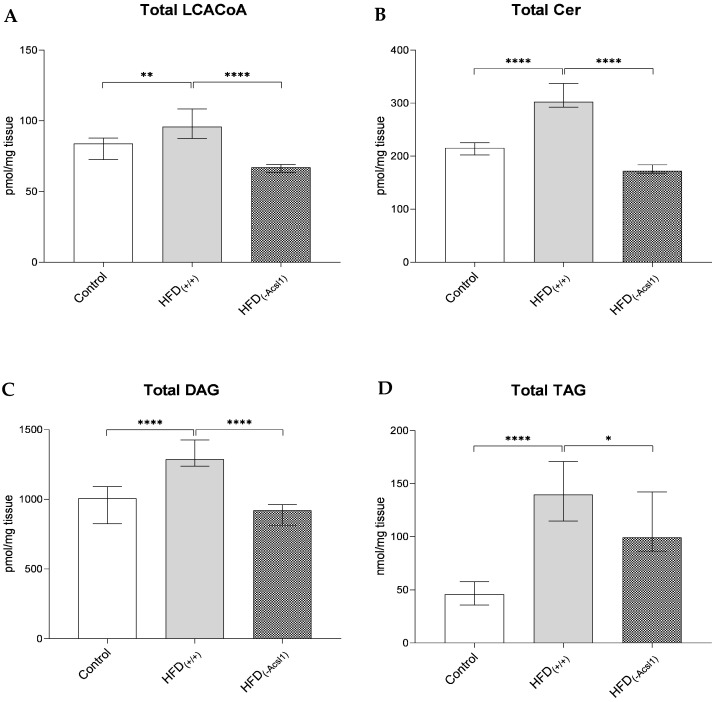
Total content of long-chain acyl-CoA (**A**), ceramides (**B**), diacylglycerols (**C**), and triacylglycerols (**D**) in the livers of control; HFD-fed (HFD_(+/+)_); and HFD-fed, ACSL1-silenced (HFD_(ACSL1−)_) mice. Bars represent medians +/− interquartile range; *—*p* < 0.05; **—*p* < 0.01; ****—*p* < 0.0001.

The high-fat diet feeding in HFD_(+/+)_ animals increased the plasma FFA concentration by 121% vs. the control value (*p* < 0.05) (Table 1). It was accompanied by an elevation in the hepatic LCACoA content (by 20%, *p* < 0.01, Table 2, Figure 2A) and all the measured lipid species. The contents of ceramide and DAG in the livers of the HFD-fed mice were higher by 48% (*p* < 0.0001) and 37% (*p* < 0.0001), respectively, vs. the control (Table 3 and Table 4, Figure 2B,C). Hepatic TAG was most affected by HFD consumption, with the levels in the liver of the HFD_(+/+)_ mice being almost three times higher than in the control (Figure 2D). Regarding individual molecular species, the content of ceramides with oleoyl- (d18:1/C18:1), stearoyl- (d18:1/C18:0), arachidoyl (d18:1/C20:0), and behenoyl-ceramide (d18:1/C22:0) was affected the most under HFD consumption (Table 3). The same was noted for DAG species, which contained 18-carbon chain fatty acids (C16:0/18:0-DAG, C18:0/18:0-DAG, C18:0/18:1-DAG and C18:0/18:2-DAG; Table 4).

The ACSL1 silencing in HFD-fed mice contributed to a slight decrease in the plasma FFA compared to the values in their non-silenced HFD_(+/+)_ counterparts (by 15%, *p* < 0.05; Table 1). A similar, yet greater, change was observed at the hepatic level in LCACoA, which decreased by 32% (*p* < 0.0001) under ACSL1 silencing (Table 1, Figure 2A). The largest decreases were noticed for C16:0-CoA, C18:0-CoA, and C18:1-CoAs. As far as the ceramide and DAG content was concerned, ACSL1 silencing in the livers of HFD_(−ACSL1)_ mice significantly decreased the hepatic content of both Cer and DAG by 44% and 33%, respectively, vs. HFD_(+/+)_ values (*p* < 0.0001 in both cases; Figure 2B,C). Hepatic TAG displayed a similar degree of down-regulation (by 24%, *p* < 0.05, Figure 2D). The most affected ceramide molecular species were d18:1/C18:1, d18:1/C18:0, d18:1/C20:0, d18:1/C22:0, d18:1/C24:1, and d18:1/C24:0 (Table 3), whereas the reduction in hepatic DAG was strongly visible for C16:0/C16:0, C16:0/C18:1, C18:2/C18:2, C18:1/C18:1, and C18:0/C18:0 diacylglycerols (Table 4).

Our results show that the HFD feeding led to an increase in the plasma FFA and subsequent accumulation of both the signaling and neutral lipid classes, whereas the ACSL1 silencing was effective in terms of hindering the accretion of FFA towards hepatic lipids in the HFD-fed C57BL/6J mice.

### 3.3. Hepatic Response to Insulin

**Figure 3:**
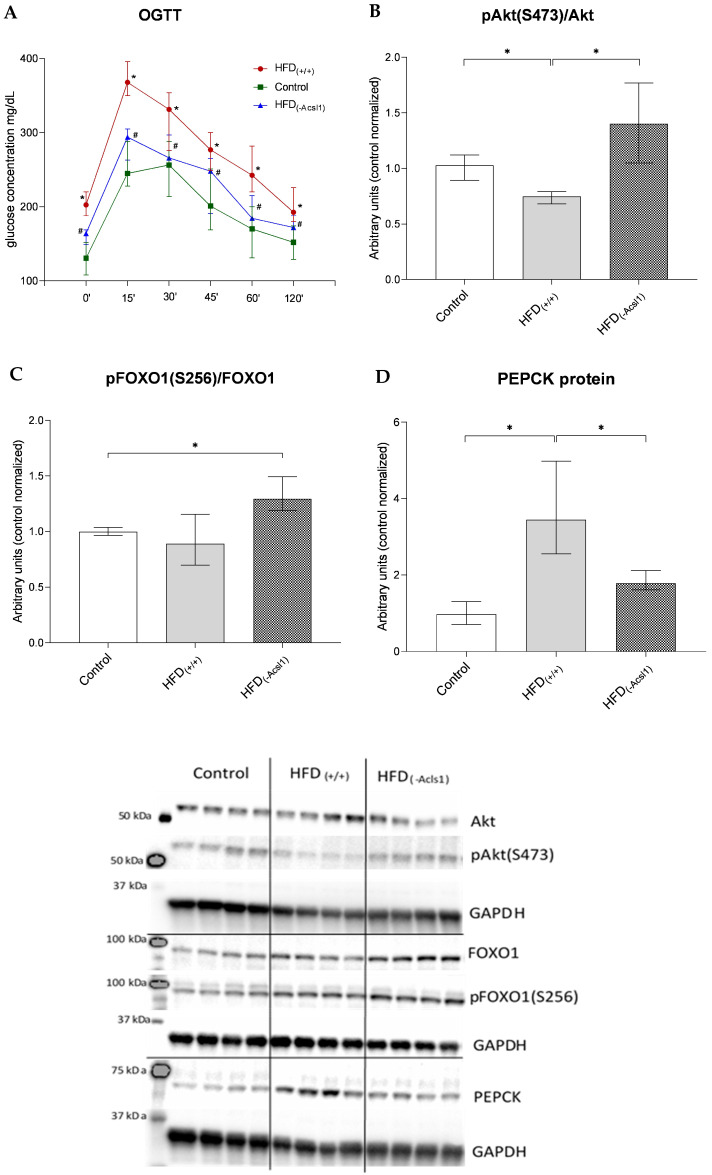
The impact of the HFD consumption and hepatic Acsl1 gene silencing on plasma glucose profile during the OGTT (**A**); the phosphorylation of PKB/Akt (pAKT S473) (**B**); the serine phosphorylation of FOXO1 (pFOXO1 S256) (**C**); and the PEPCK level (**D**). Bars represent medians +/− interquartile range; *—*p* < 0.05; Panel (**A**): #—*p* < 0.05 vs. HFD_(+/+)_.

Both the Cer and DAG hepatic accumulation under HFD consumption coincided with down-regulation of insulin-stimulated Akt phosphorylation at the Ser473 moiety (by 27%, *p* < 0.05 vs. control, Figure 3B). A similar trend was observed for the FOXO1 phosphorylation status, yet the change did not reach any significance threshold (Figure 3C). The protein expression of PEPCK, a key regulator of hepatic gluconeogenesis, increased by approx. 3.6-fold (*p* < 0.05) in the hepatic tissue of the HFD_(+/+)_ animals vs. the values observed in LFD-fed control mice (Figure 3D).

The decrease in hepatic ACSL1 expression in the HFD_(−ACSL1)_ mice rescued insulin-stimulated Akt phosphorylation (up-regulation by 88%) and significantly decreased liver PEPCK expression (down-regulation by 48% vs. HFD_(+/+)_, *p* < 0.05 in both cases; Figure 3B,D). Although the ACSL1 silencing resulted in an increase in FOXO1 phosphorylation as compared to the HFD_(+/+)_ group, it did not reach any statistical significance (Figure 3C).

The presented data indicate that the accumulation of hepatic ceramide and DAG in the HFD-fed animals was accompanied by a decrease in liver insulin sensitivity and an up-regulation of key gluconeogenic enzymes. The hepatic ACSL1 silencing in the HFD_(+/+)_ group contributed to ameliorating the adverse impact of lipid accumulation on the hepatic insulin signaling and gluconeogenic enzyme expression.

### 3.4. Hepatic Glucose Metabolism and Indices of Insulin Resistance

**Figure 4:**
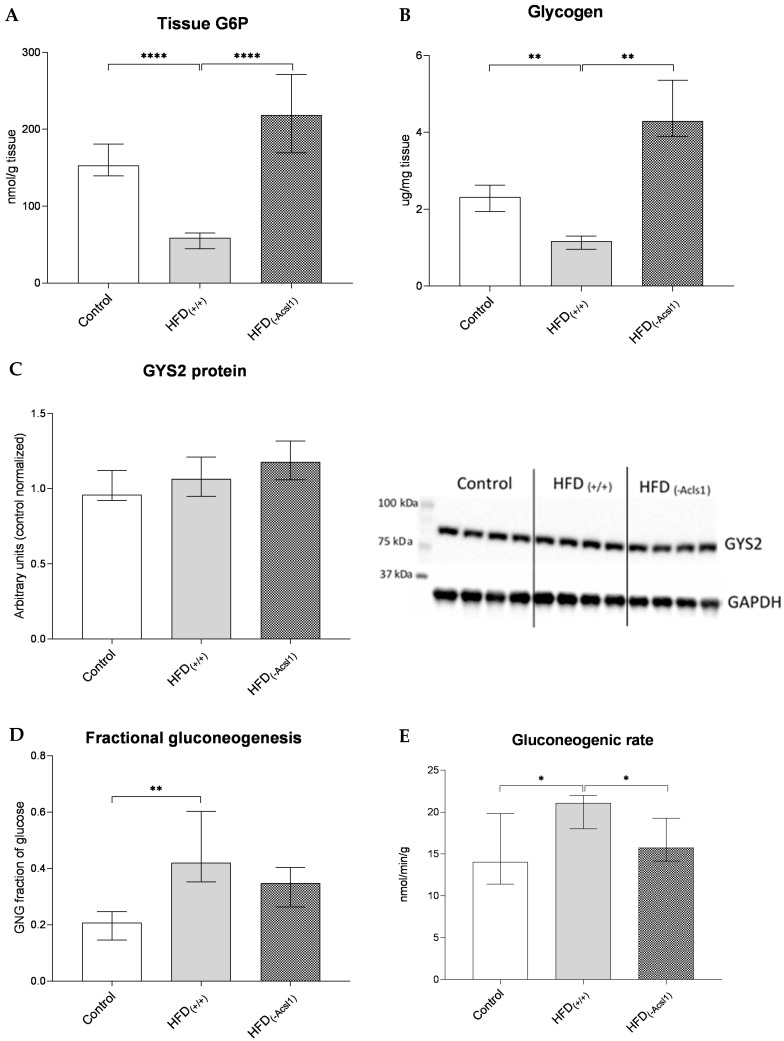
The content of liver glucose-6-phosphate (**A**); hepatic glycogen (**B**); liver GYS 2 protein expression (**C**); fractional gluconeogenesis (**D**); and gluconeogenic rate (**E**) under control; HFD-fed (HFD_(+/+)_); and HFD-fed, ACSL1-silenced (HFD_(−ACSL1)_) conditions. Bars represent medians +/− interquartile ranges; *—*p* < 0.05; **—*p* < 0.01; ****—*p* < 0.0001.

The HFD-induced detrimental changes in the hepatic insulin signaling were also visible at the level of the liver G6P content, which decreased by 67% in the HFD_(+/+)_ group (*p* < 0.0001 vs. control; Figure 4A). Although the expression of glycogen synthase 2 (GYS2) was not affected by HFD feeding (Figure 4C), the insulin-stimulated accumulation of hepatic glycogen decreased by 57% in the HFD_(+/+)_ mice (*p* < 0.01 vs. control; Figure 2B). Both the fractional gluconeogenesis (fraction of blood glucose derived from hepatic gluconeogenesis) and the gluconeogenic rate increased in the HFD_(+/+)_ animals (by approx. 100% (*p* < 0.01) and 28% (*p* < 0.05), respectively) vs. the respective values in the control group (Figure 4D,E).

In the livers of mice in the HFD_(−ACSL1)_ group, the hepatic G6P content increased by more than three times (*p* < 0.0001, Figure 4A) and hepatic glycogen by almost four times (*p* < 0.01, Figure 4B) vs. the non-silenced HFD_(+/+)_ liver. Interestingly, the GYS2 protein content was not affected by the ACSL1 silencing (Figure 4C). Fractional gluconeogenesis decreased slightly (by 17%) under the ACSL1 silencing, yet the change was insignificant (vs. HFD_(+/+)_ values, Figure 4D). Contrary to the above parameter, the decrease in the gluconeogenic rate in the HFD_(−ACSL1)_ mice reached significance, being 15% lower than the values from HFD_(+/+)_ animals (*p* < 0.05, Figure 4E).

All of the above effects of both the HFD feeding and ACSL1 silencing in the HFD-fed animals significantly altered the systemic indices of IRes. The feeding with the high-fat diet increased plasma glucose and insulin concentration in the HFD_(+/+)_ mice, resulting in an increase in the HOMA-IR (vs. control, *p* < 0.05, Table 1). In addition, the HFD_(+/+)_ animals were indicative of the impaired glucose tolerance as compared to the control counterparts, as revealed by OGTT (Figure 3A).

The ACSL1 silencing under HFD consumption resulted in decreases in the glucose, insulin, and HOMA-IR values (*p* < 0.05 for all cases), as well as a significant improvement in OGTT parameters (vs. HFD_(+/+)_, Figure 3A).

## 4. Discussion

The liver plays a key in glucose homeostasis by means of both the glucose uptake from the blood in order to store it as glycogen in the postprandial state and the release of glucose from glycogenolysis and gluconeogenesis in the post-absorptive state. The liver is also crucial for the whole-body lipid metabolism. Lipid metabolism disorders in the liver have been repeatedly demonstrated to result in decreased hepatic insulin sensitivity [37,38]. This work is the first to present that the ACSL1 gene silencing in the liver decreases the hepatic content of Cer and DAG and improves the insulin signaling pathway in animals with HFD-induced InsR. Previous works focusing on the silencing or knock-out of the ACSL1 gene in the livers of animals fed a Western diet have not analyzed all the biologically active lipids that we have measured. The work by Singh et al. examined the effect of ACSL1 silencing in the liver on bile acid synthesis in animals fed a Western diet [39]. In the aforementioned study, the silencing of the ACSL1 gene in the liver was found not to affect the plasma fatty acid concentrations, but surprisingly, it triggered a slight, but significant, increase in the liver TAG levels. Although, in our study, we focused on the lipids implicated in the induction of insulin resistance, we also estimated the plasma content of fatty acids at the level of liver TAG. In our work, we noted that the silencing of ACSL1 in the liver under HFD consumption slightly, but significantly, lowered the plasma FFA concentrations and triggered a decrease in the contents of all hepatic lipids, including TAG. However, it is worth noting that the animals in our experiment consumed a lipid-rich diet, in which 60% of the calories came from fats, while in the aforementioned study, the animals consumed a Western-type diet, with approx. 45% of calories in form of fat. Moreover, the other reason for the discrepancy between the aforementioned results and our results may be a different method of silencing the ACSL1 gene. In the work by Singh et al. [39], the silencing of the ACSL1 gene was performed by means of adenovirus infection, which was associated with an intensified immune system response, while in our work, we used the HGD method. Another study used liver-specific ACSL1 knockout animals housed for three months on a Western diet [5]. Despite the observed decrease in the LCACoA levels in the knockout mice, no decrease in the DAG levels was observed, except for TAG, which was the case that proved the increase in the content of those lipids. In our work, we found the levels of all the lipids measured in this study to have increased in the livers of animals fed an HFD. Those data are consistent with those previously obtained by our team [7,8], as well as with the data obtained by other researchers [40,41,42,43,44]. The increase in the content of hepatic lipids overlapped with the elevation in the HOMA-IR value, a weakened response to glucose load, and inhibition of the insulin pathway, manifesting in decreased Akt/PKB phosphorylation and increased protein expression of PEPCK, the rate-limiting enzyme in gluconeogenesis (Figure 3). The elevated PEPCK content suggested an increased rate of glucose production, which was confirmed by examining the rate of gluconeogenesis through glucose MIDA analysis (mass isotopomer distribution analysis). An increase in hepatic glucose output is a feature of T2D that is commonly attributed to gluconeogenesis rather than glycogenolysis [45,46]. In our work, in the HFD_(+/+)_ group, we observed not only an increase in the rate of gluconeogenesis, but also an inhibition of glycogen synthesis, which translated into a decrease in the glycogen content, although the content of GYS2, the enzyme responsible for glycogen synthesis, was not affected. It is plausible to state that the most effective GYS2 activator is the G6P. The content of G6P in the liver of HFD_(+/+)_ animals was significantly lower compared to other groups, which may justify a decrease in GYS2 enzymatic activity and a reduction in hepatic glycogen levels. Similar decreases in the liver glycogen content in insulin-resistant humans and animals have also been observed by other researchers [46,47]. Both increased gluconeogenesis and decreased glycogen synthesis contribute to increases in plasma glucose concentration. The silencing of the ACSL1 gene in the livers of the HFD-fed mice led to decreases in all the measured lipid levels as compared to the HFD_(+/+)_ group. ACSL1, as the major isoform of ACSL in the liver, is responsible for the availability of the substrate, acyl-CoA, for the de novo synthesis of intracellular lipids. The decrease in the hepatic content of Cer and DAG coincided with an augmentation of the insulin sensitivity at both the systemic and hepatic levels, which manifested as an improved response to glucose load and liver insulin signaling. The level of tissue augmentation of the insulin pathway was visible as an up-regulation of Akt/PKB phosphorylation, as well as by the increase in the inhibitory phosphorylation of the FOXO1 protein as compared to the HFD_(+/+)_ animals. Phosphorylation of FOXO1 blocks its transcriptional activity and thus inhibits the expression of gluconeogenic genes, as confirmed by the decrease in the PEPCK protein expression in the livers of the HFD_(−ACSL1)_ mice as compared to the HFD_(+/+)_ animals. The changes observed in the ACSL1-silenced liver associated with FOXO1 phosphorylation and PEPCK levels translated into a decrease in the rate of gluconeogenesis. Hepatic glycogen synthesis is another important process regulated by insulin. The enzyme directly responsible for the synthesis of glycogen in the liver is GYS2. Interestingly, we did not observe any significant differences in the protein expression of the GYS2 enzyme between the studied groups. However, since G6P is an important allosteric activator of GYS2 [48], an increase in the hepatic level of G6P may also lead to GYS2 activation. This allosteric effect was likely responsible for the accumulation of liver glycogen in the ACSL1-silenced animals, which concurrently displayed significantly higher levels of G6P as compared to the non-silenced HFD-fed counterparts. Our study clearly indicates a significant contribution of ACSL1 to the accumulation of biologically active lipids. Ceramides synthesized de novo from ACSL1-derived acyl-CoAs seem to play an especially key role in the deterioration of the functioning of the insulin pathway in the liver. This results in impaired regulation of gluconeogenesis and glycogenesis, as well as an increase in blood glucose concentration. Consumption of the HFD diet triggered increases in the levels of all the studied lipid groups, and ACSL1 liver-specific gene silencing was able to significantly attenuate this effect. However, the greatest variation was noted in the case of ceramides. Moreover, the ACSL1 silencing resulted in improved functioning of the insulin pathway, primarily at the level of Akt protein phosphorylation. Weakened phosphorylation of that protein often results from ceramide accumulation. The improved functioning of the insulin pathway at this stage is probably an effect of the reduced ceramide content. It can therefore be concluded that the hepatic ceramide pool is more dependent on the ACSL1-supplied acyl-CoAs than the DAG pool.

Although we performed a detailed analysis of LCA-CoA, ceramide, and DAG composition using the LC/MS/MS technique, the study would also benefit from a wider approach to lipid analysis. This is justified by the possible significant impact of ACSL1 silencing on all the cellular lipid classes. Performing non-targeted or semi-targeted lipidomic analysis would reveal the impact of ACSL1 silencing on a wider range of lipid species that are possibly affected in the greater extent. The major rationale behind our study was to observe the effects of ACSL1 silencing on lipids with proven signaling properties crucial for the induction of lipid resistance. Thus, lipidomic analysis of all identifiable lipid species in the ACSL1 silenced livers of HFD-fed animals warrants its own separate study.

## 5. Conclusions

In summary, our studies show that the inhibition of LCACoA production can significantly contribute to improving the function of the hepatic insulin pathway and, thus, normalizing glucose metabolism under HFD-induced InsR conditions, which is probably the result of a decrease in both the ceramide and DAG content. It should be noted, however, that ACSL1 activity in the liver determines the level of ceramides to a greater extent than diacylglycerols.

## Figures and Tables

**Table 1:** The impact of the high-fat diet and Acsl1 liver silencing on the indices of insulin resistance and plasma FFA.

	Control	HFD_(+/+)_	HFD_(−Acsl1)_
Fasting glucose (mg/dL) (*n* = 10)	130.5 (109.5–149.8)	202.5 (191.8–217.8) *	182.5 (167.8–189.0) ^#^
Fasting insulin (μIU/mL) (*n* = 10)	7.15 (5.60–11.33)	25.25 (13.33–33.30) *	15.00 (11.10–16.73) ^#^
HOMA-IR (*n* = 10)	0.40 (0.200–0.700)	2.25 (1.08–2.58) *	1.00 (0.88–1.33) ^#^
Total FFAs (nmol/mL) (*n* = 10)	220.9 (195.0–232.5)	488.3 (465.3–521.6) *	415.3 (382.4–446.7) ^#^
Baseline body (g) (*n* = 10)	17.45 (16.70–18.73)	18.25 (17.48–9.13)	19.53 (18.7–20.04)
End-point body weights (g) (*n* = 10)	27.18 (26.64–28.36)	30.75 (28.89–34.15)	32.40 (30.64–35.53)

Data expressed as medians and interquartile range; *: *p* < 0.05 vs. control, #: *p* < 0.05 vs. HFD_(+/+)_.

**Table 2:** Hepatic content of individual of Acyl-CoA molecular species under *Acls1* silencing in mouse livers.

LCACoA	Control	HFD_(+/+)_	HFD_(−Acsl1)_
C14:0	1.55 (1.38–1.75)	1.93 (1.70–2.27) *	1.50 (1.40–1.67) ^#^
C16:0	9.44 (8.71–10.90)	16.32 (13.12–17.74) *	9.03 (8.17–11.00) ^#^
C16:1	10.37 (9.00–11.30)	3.93 (3.51–4.44) *	3.18 (2.68–3.39) ^#^
C18:0	13.72 (11.68–14.36)	18.85 (13.32–19.84) *	10.16 (9.20–11.12) ^#^
C18:1	15.20 (13.81–17.83)	18.01 (13.41–18.79)	10.59 (9.11–10.92) ^#^
C18:2	29.65 (27.34–33.50)	38.39 (35.45–44.53) *	31.37 (28.38–33.41) ^#^
C20:0	1.44 (1.05–1.51)	1.64 (1.44–1.78)	1.23 (1.11–1.36) ^#^

Data expressed as medians (pmol/mg tissue) and interquartile ranges; *n* = 10 per control and HFD_(+/+)_ group, *n* = 13 per HFD_(−Acsl1)_ group; *: *p* < 0.05 vs. control, #: *p* < 0.05 vs. HFD_(+/+)_.

**Table 3:** The contents of ceramide molecular species in the in the *Acls1*-silenced mouse livers.

Ceramide	Control	HFD_(+/+)_	HFD_(−Acsl1)_
d18:1/C14:0	0.043 (0.035–0.049)	0.038 (0.034–0.041)	0.031 (0.029–0.033) ^#^
d18:1/C16:0	6.83 (6.56–7.66)	7.14 (6.89–7.54)	7.25 (6.89–7.81)
d18:1/C18:0	1.50 (1.36–1.62)	3.28 (2.80–3.57) *	2.26 (2.00–2.26) ^#^
d18:1/C18:1	0.148 (0.136–0.173)	0.2817 (0.267–0.316) *	0.212 (0.192–0.233) ^#^
d18:1/C20:0	4.24 (3.91–4.83)	12.48 (11.66–13.61) *	9.82 (8.21–10.62) ^#^
d18:1/C22:0	75.42 (60.19–82.90)	133.8 (129.5–176.0) *	72.40 (64.88–80.42) ^#^
d18:1/C24:0	45.97 (41.58–51.69)	51.24 (47.99–53.49)	32.04 (31.22–34.04) ^#^
d18:1/C24:1	80.60 (74.60–84.78)	87.24 (85.16–91.60) *	47.93 (43.14–55.22) ^#^

Data expressed as medians (pmol/mg tissue) and interquartile ranges; *n* = 10 per control and HFD_(+/+)_ group, *n* = 13 per HFD_(−Acsl1)_ group; *: *p* < 0.05 vs. control, #: *p* < 0.05 vs. HFD_(+/+)_.

**Table 4:** The contents of DAG molecular species in the ACSL1-silenced mouse livers.

Diacylglycerol	Control	HFD_(+/+)_	HFD_(−Acsl1)_
C14:0/14:0	0.66 (0.58–0.71)	0.58 (0.51–0.69)	0.61(0.49–0.70)
C16:0/16:0	16.65 (12.76–17.50)	19.48 (17.67–26.94) *	8.17 (6.99–10.04) ^#^
C16:0/18:0	3.670 (3.50–4.37)	10.08 (8.88–11.65) *	9.23 (7.82–10.57)
C16:0/18:1	395.3 (305.7–446.8)	513.5 (460.2–576.3) *	273.6 (217.7–335.2) ^#^
C16:0/18:2	182.3 (166.1–207.7)	229.7 (210.4–251.4) *	177.1 (160.6–203.4) ^#^
C18:0/18:0	1.76 (1.62–2.06)	3.81 (3.12–4.22) *	2.71 (2.53–3.40) ^#^
C18:0/18:1	17.79 (15.82–19.33)	42.85 (39.36–48.57) *	35.28 (31.85–37.41) ^#^
C18:0/18:2	16.40 (11.92–19.91)	40.90 (36.92–52.96) *	35.01 (32.44–39.59) ^#^
C18:1/18:1	258.0 (194.2–300.9)	292.5 (274.3–315.2)	223.5 (196.6–254.9) ^#^
C18:2/18:2	73.13 (66.38–77.92)	105.0 (84.48–108.5) *	76.21 (63.68–81.41) ^#^
C18:0/20:4	30.23 (28.20–33.92)	49.47 (40.20–54.59) *	39.28 (34.90–44.30) ^#^

Data expressed as medians (pmol/mg tissue) and interquartile ranges; *n* = 10 per control and HFD_(+/+)_ group, *n* = 13 per HFD_(−Acsl1)_ group; *: *p* < 0.05 vs. control, #: *p* < 0.05 vs. HFD_(+/+)_.

## Data Availability

Data are available at: https://osf.io/dq9pw/?view_only=c5bd6a2ee35b468fa42de1d74bd1873e, accessed on 8 February 2024.

## Supplementary Materials

The following supporting information can be downloaded at: https://www.mdpi.com/article/10.3390/nu16071003/s1, Figure S1: Original blot of Acsl1, Figure S2: Original blot of Akt, Figure S3: Original blot of pAKT(S473), Figure S4: Original blot of FOXO1, Figure S5: Original blot of pFOXO1(S256), Figure S6: Original blot of PEPCK, Figure S7: Original blot of GYS2, Figure S8: Original blot of GAPDH.
