# Supplementary material for: The Role of Acyl-CoA Synthetase 1 in Bioactive Lipid Accumulation and the Development of Hepatic Insulin Resistance
Source: Nutrients. 2024 Mar 29;16(7):1003. doi: 10.3390/nu16071003 (PMC11013895; doi:10.3390/nu16071003)

Supplementary Figures

The sequence of application of the samples into the gel wells was individually set for particular proteins.

Figure S1. Full unedited gel for Figure Acsl1**. The sequence of application the samples into the gel wells:** 1 lane – Protein standard (STD), 2-6 lane –Control sample, 7-11 lane – HFD $_{(+/+)}$ sample, 20-24 lane – HFD$_{(-ACSL1)}$ sample, 25 lane – Protein standard (STD), the rest of the lane – other samples (not analyzed - X).

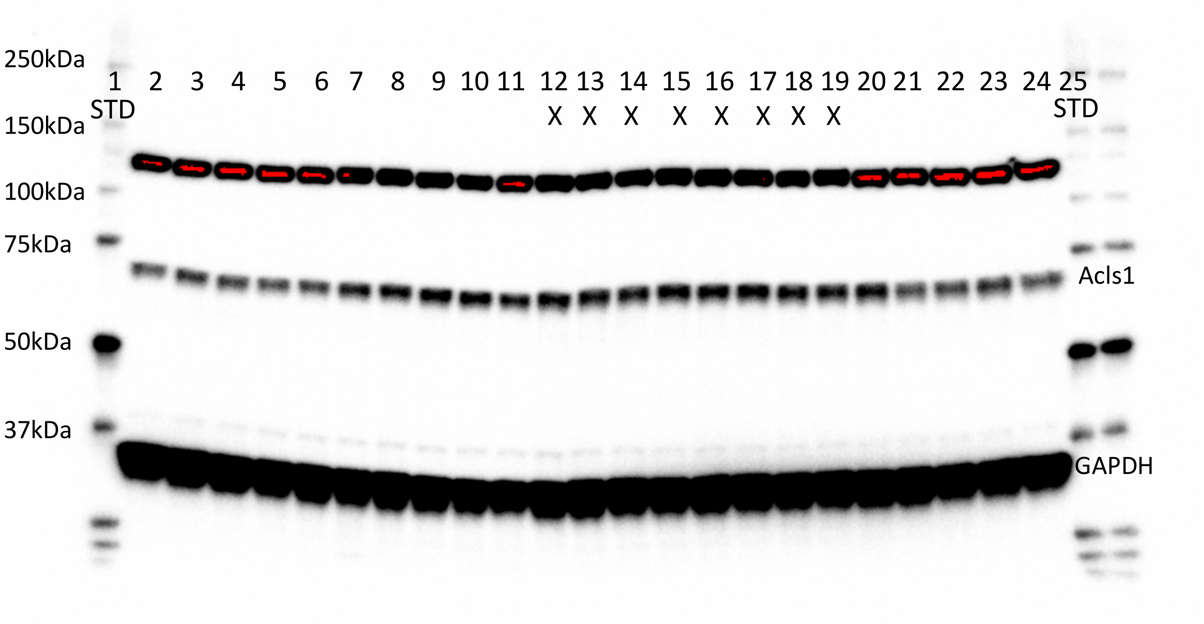

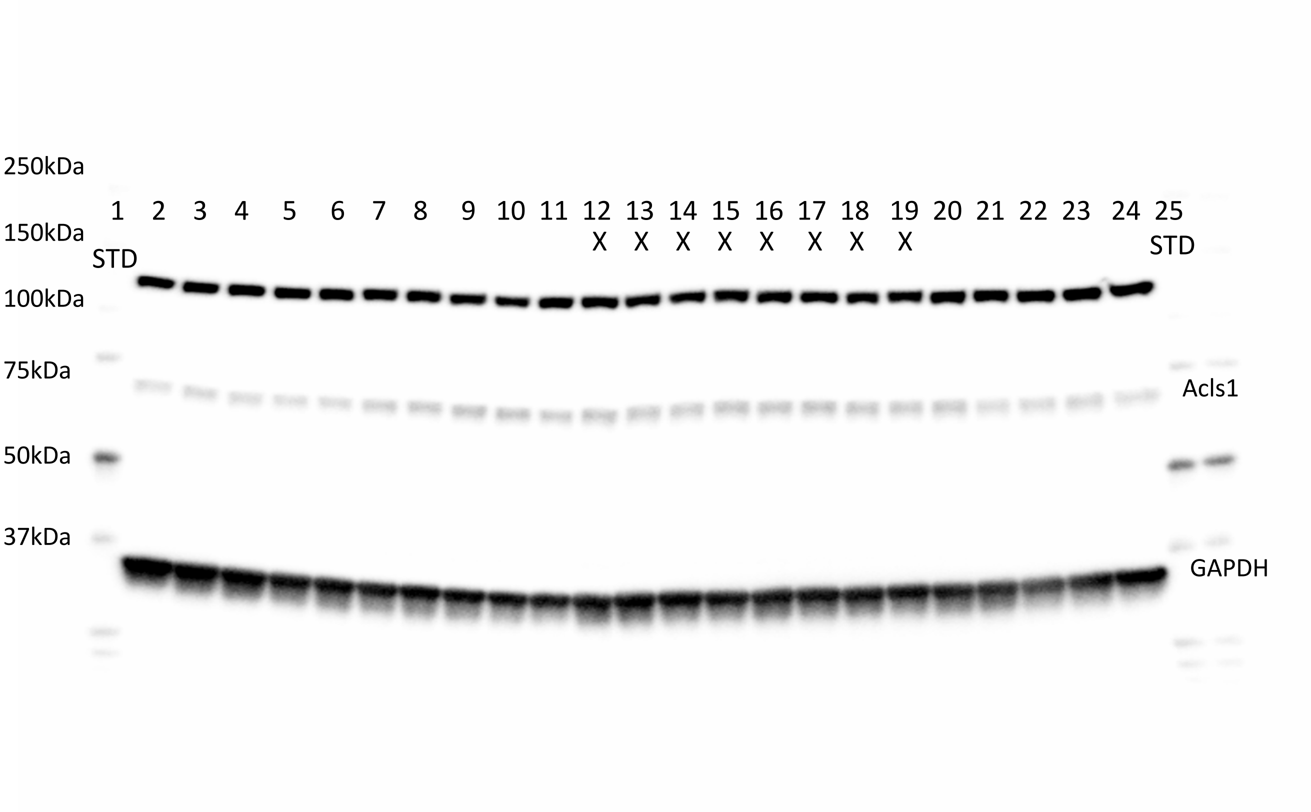

Figure S2. Full unedited gel for Figure Akt. **The sequence of application the samples into the gel wells:** 1 lane – Protein standard (STD), 2-5 lane – Control sample, 6-9 lane – HFD $_{(+/+)}$ sample, 10-13 lane – HFD$_{(-ACSL1)}$ sample, 26 lane – Protein standard (STD), the rest of the lane – other samples (not analyzed - X). *GAPDH used for normalization was run on a different gel (pAkt(S473) gel nr 3) and is located below.*

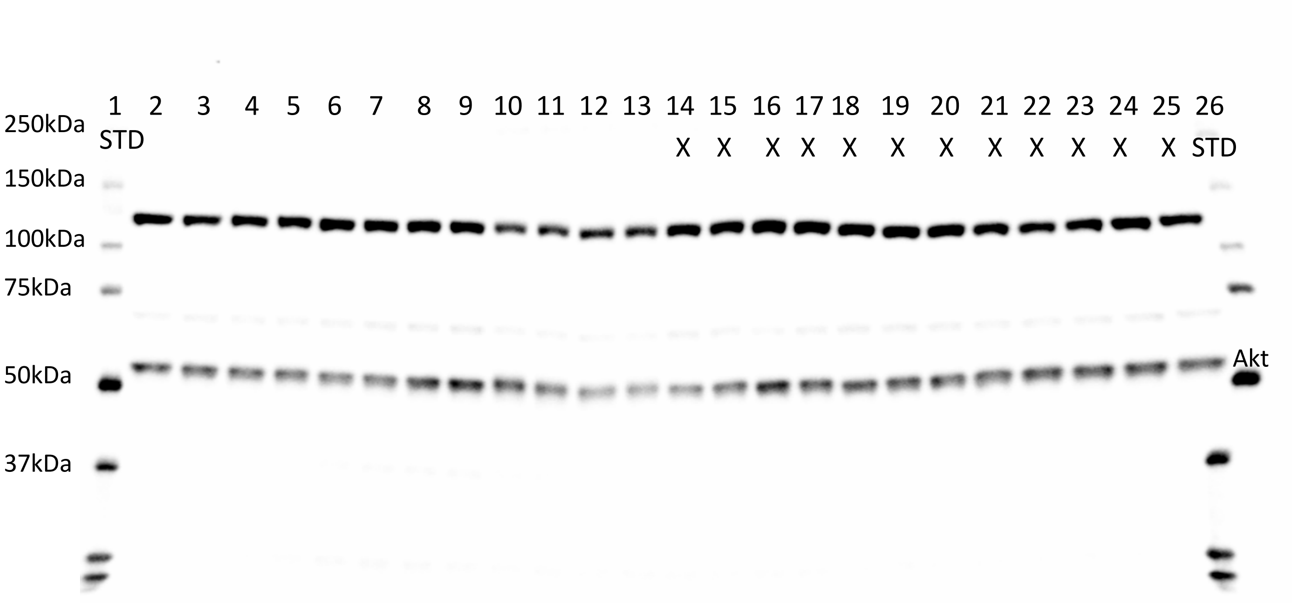

Figure S3. Full unedited gel for Figure pAkt(S473). **The sequence of application the samples into the gel wells:** 1 lane – Protein standard (STD)**,** 2-5 lane – Control sample, 6-9 lane – HFD $_{(+/+)}$ sample, 10-13 lane – HFD$_{(-ACSL1)}$ sample, 26 lane – Protein standard (STD), the rest of the lane – other samples (not analyzed - X).

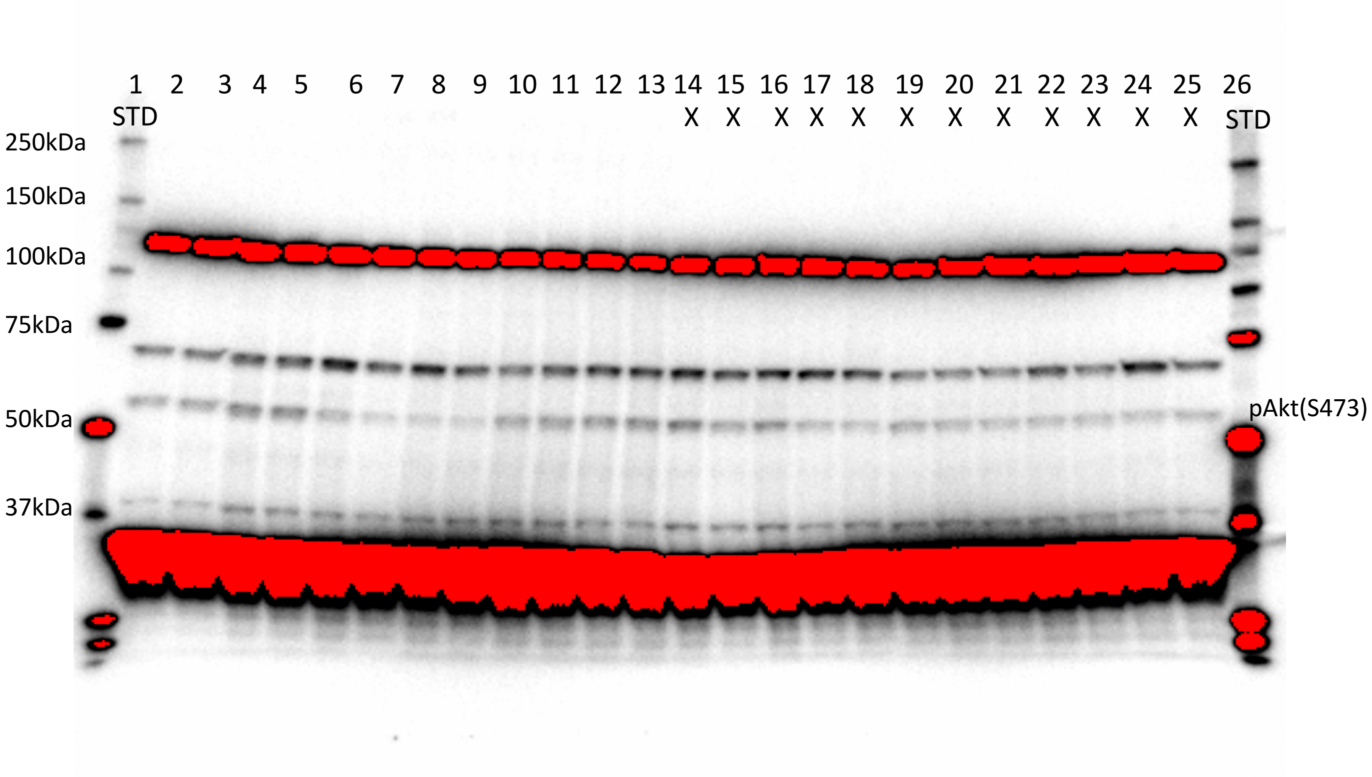

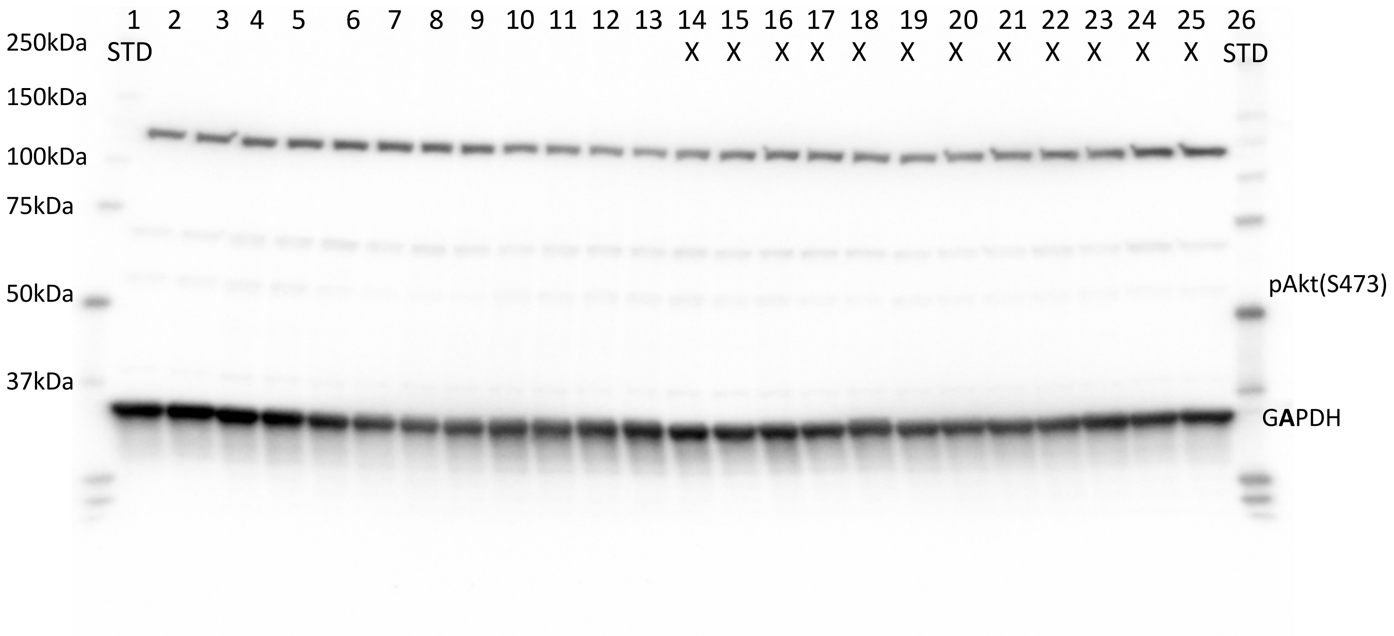

Figure S4. Full unedited gel for Figure FOXO1. **The sequence of application the samples into the gel wells:** 1 lane – Protein standard (STD)**,** 2-5 lane – Control sample, 6-9 lane – HFD $_{(+/+)}$ sample, 10-13 lane – HFD$_{(-ACSL1)}$ sample, 26 lane – Protein standard (STD), the rest of the lane – other samples (not analyzed - X). *GAPDH used for normalization was run on a different gel (gel nr 8) and is located below.*

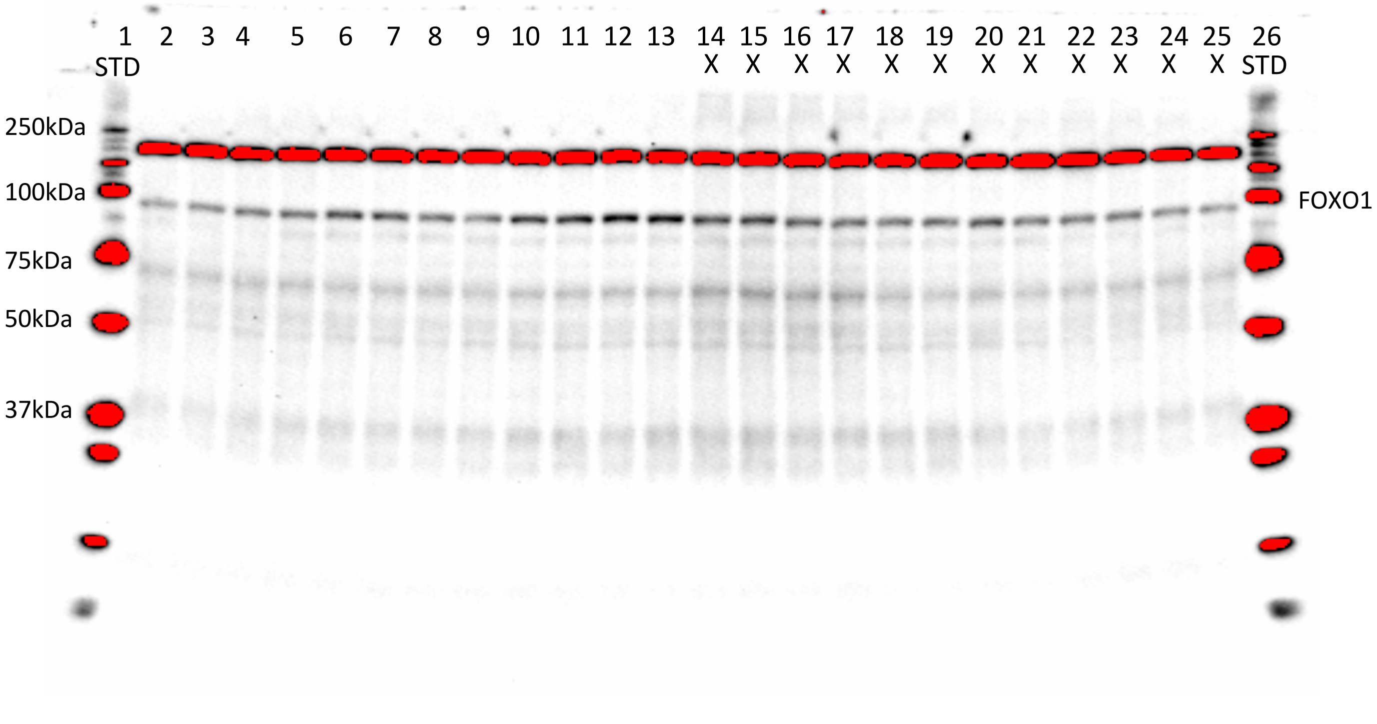

Figure S5. Full unedited gel for Figure FOXO1 (S256). **The sequence of application the samples into the gel wells:** 1 lane – Protein standard (STD), 2-5 lane – Control sample, 6-9 lane – HFD $_{(+/+)}$ sample, 10-13 lane – HFD$_{(-ACSL1)}$ sample, 26 lane – Protein standard (STD), the rest of the lane – other samples (not analyzed - X). *GAPDH used for normalization was run on a different gel (gel nr 8) and is located below.*

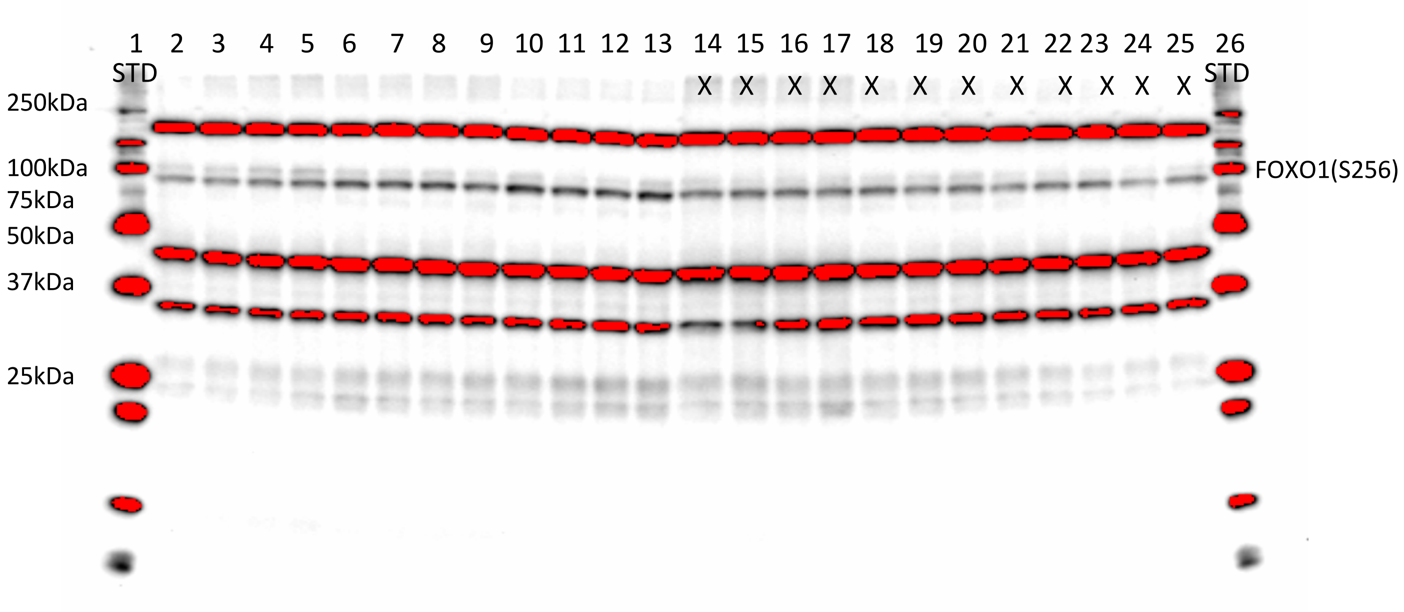

Figure S6. Full unedited gel for Figure PEPCK. **The sequence of application the samples into the gel wells:** 1 lane – Protein standard (STD)**,** 2-5 lane – Control sample, 6-9 lane – HFD $_{(+/+)}$ sample, 10-13 lane – HFD$_{(-ACSL1)}$ sample, 26 lane – Protein standard (STD), the rest of the lane – other samples (not analyzed - X).

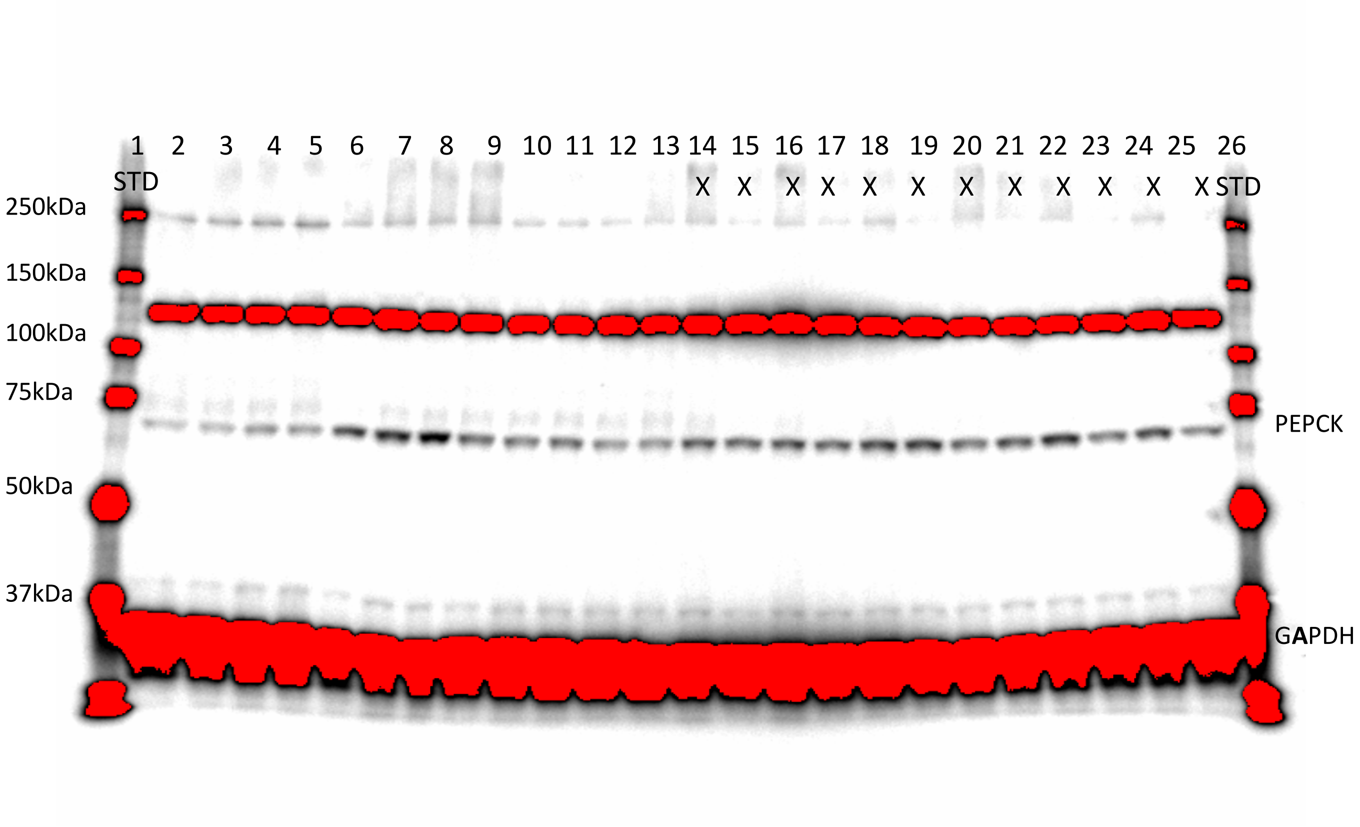

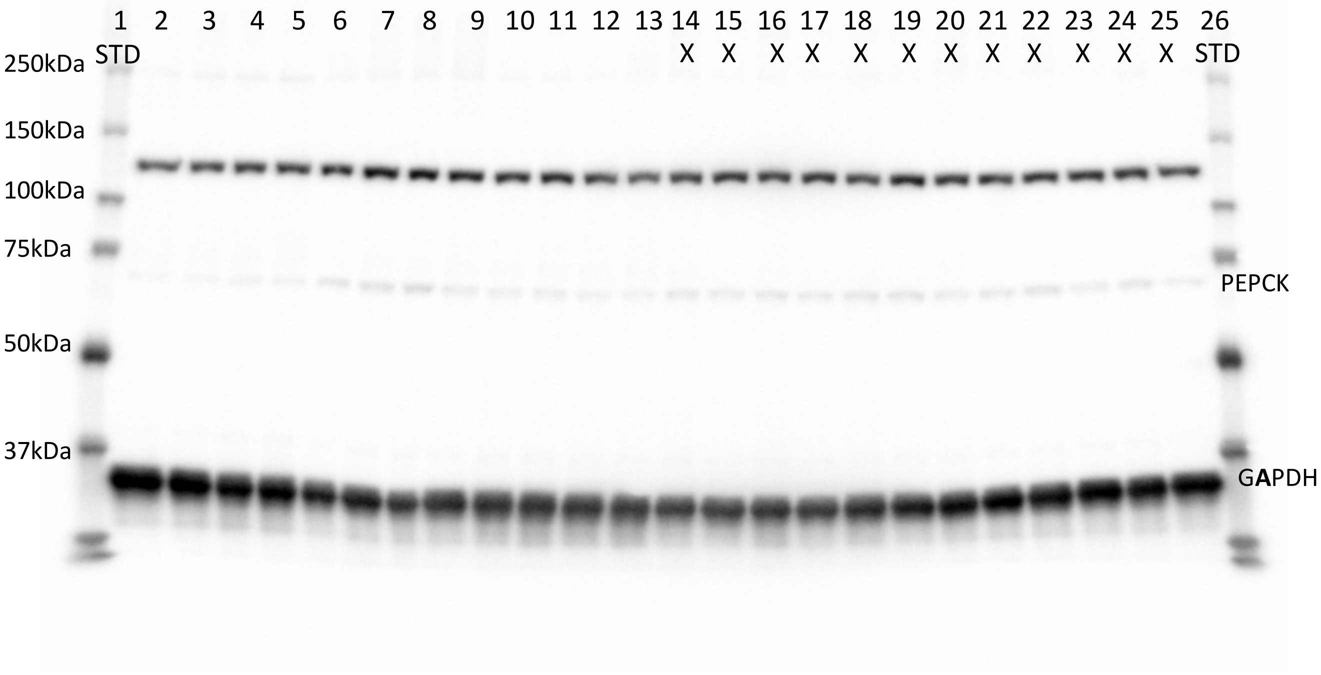

Figure S7. Full unedited gel for Figure GYS2. **The sequence of application the samples into the gel wells:** 1 lane – Protein standard (STD), 2-5 lane – Control sample, 6-9 lane – HFD $_{(+/+)}$ sample, 10-13 lane – HFD$_{(-ACSL1)}$ sample, 26 lane – Protein standard (STD), the rest of the lane – other samples (not analyzed). *GAPDH used for normalization was run on a different gel (gel nr 8) and is located below.*

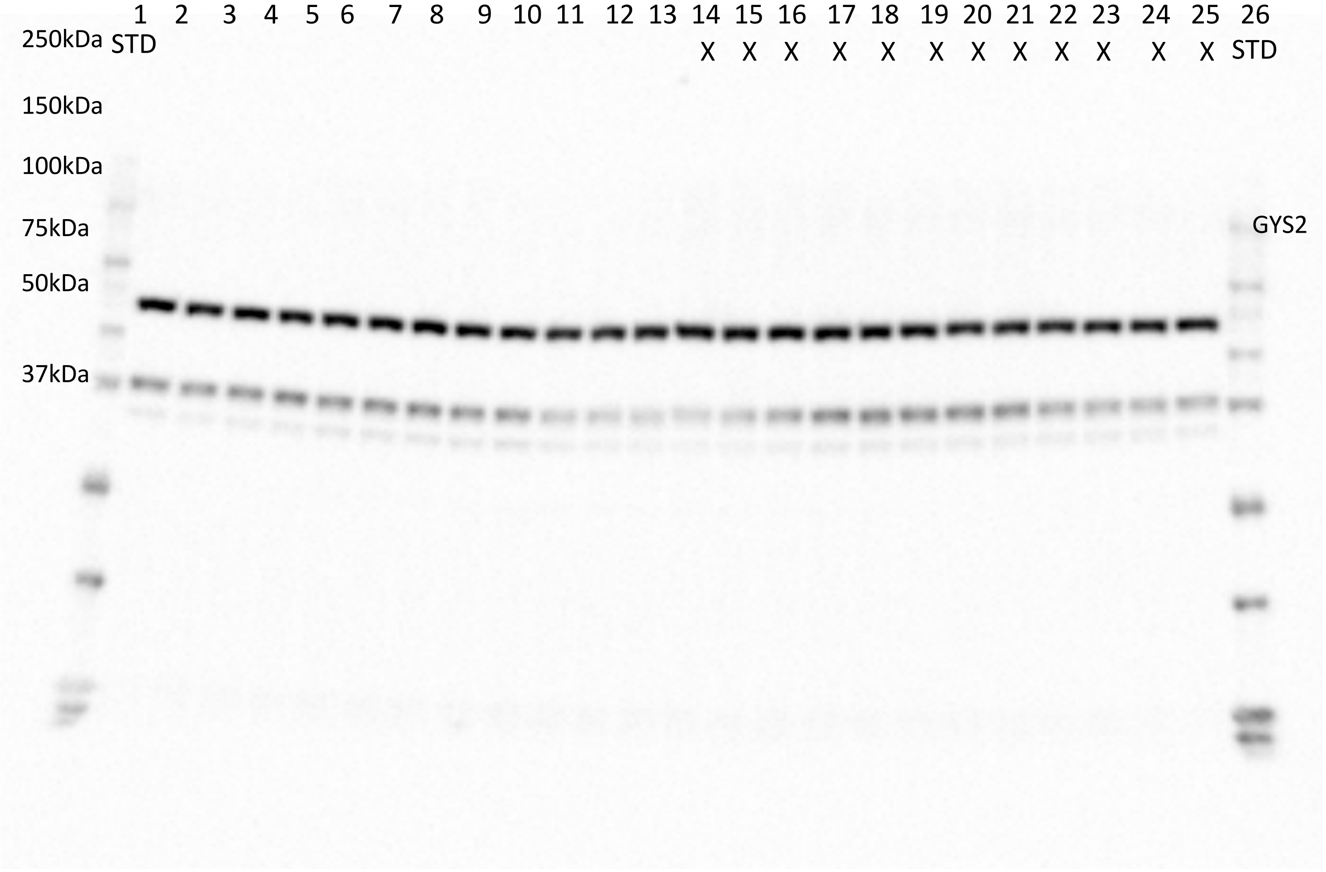

Figure S8. Full unedited gel for Figure FOXO1, pFOXO1(S256) and GYS2. **The sequence of application the samples into the gel wells:** 1 lane – Protein standard (STD), 2-5 lane – Control sample, 6-9 lane – HFD $_{(+/+)}$ sample, 10-13 lane – HFD$_{(-ACSL1)}$ sample, 26 lane – Protein standard (STD), the rest of the lane – other samples (not analyzed - X).

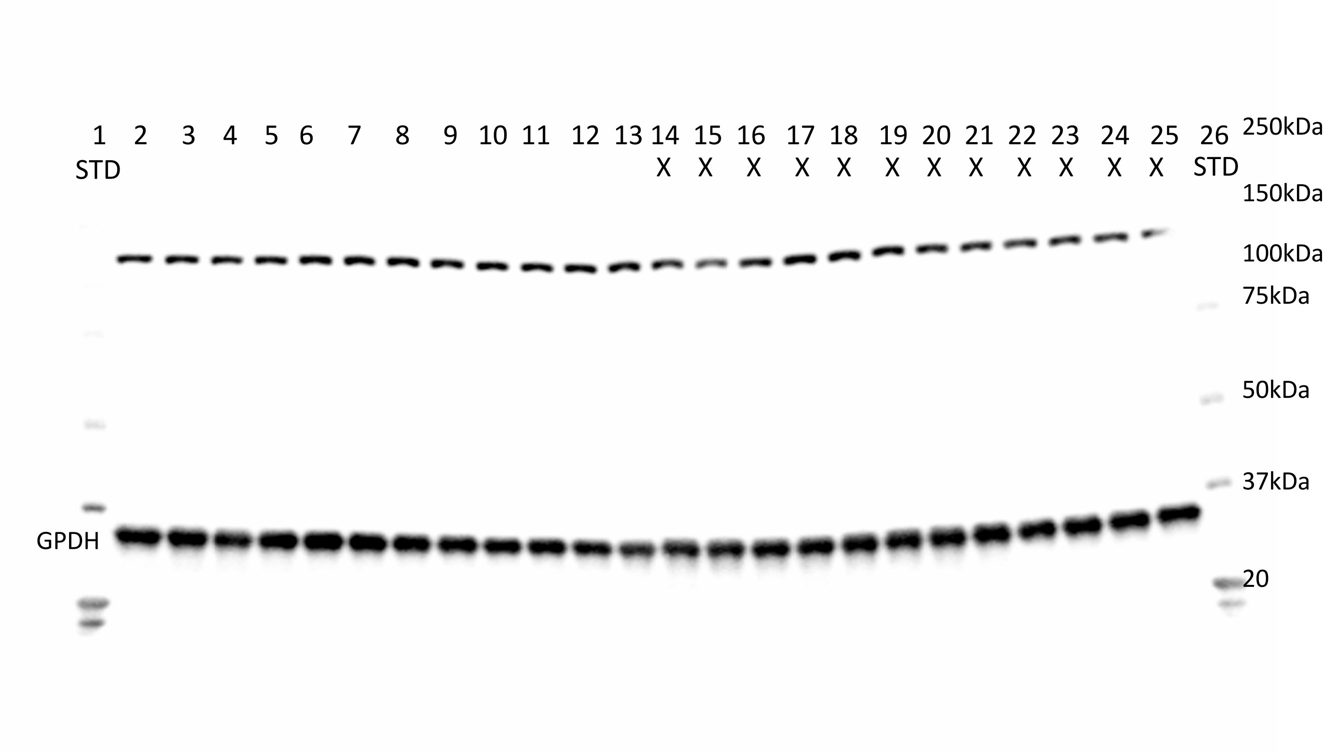

Supplement: Supplementary file 1 [file nutrients-16-01003-s001.zip › nutrients-2889848-supplementary.pdf]
